# Supplementary material for: Pressure-induced emission of cesium lead halide perovskite nanocrystals
Source: Nat Commun. 2018 Oct 29;9:4506. doi: 10.1038/s41467-018-06840-8 (PMC6206024; doi:10.1038/s41467-018-06840-8)
Supplement: Supplementary file 1 — Supplementary Information [file 41467_2018_6840_MOESM1_ESM.pdf]

Supplementary Information for

Pressure-Induced Emission of Cesium Lead Halide Perovskite Nanocrystals

Zhiwei Ma,[1] Zhun Liu,[2] Siyu Lu,[3] Lingrui Wang,[1] Xiaolei Feng,[4] Dongwen Yang,[2] Kai Wang,[1] Guanjun Xiao,[1,]* Lijun Zhang,[2,1]* Simon A. T. Redfern,[4] and Bo Zou[1,]*

[1]State Key Laboratory of Superhard Materials, College of Physics, Jilin University Changchun 130012, China

[2]Key Laboratory of Automobile Materials of MOE, and School of Materials Science, Jilin University, Changchun 130012, China

[3]College of Chemistry and Molecular Engineering, Zhengzhou University Zhengzhou 450001, China

[4]Department of Earth Sciences, Downing Street, University of Cambridge, Cambridge, CB2 3EQ, UK

*Corresponding author. Email: xguanjun@jlu.edu.cn (G.X.); lijun_zhang@jlu.edu.cn (L.Z); zoubo@jlu.edu.cn (B.Z.)

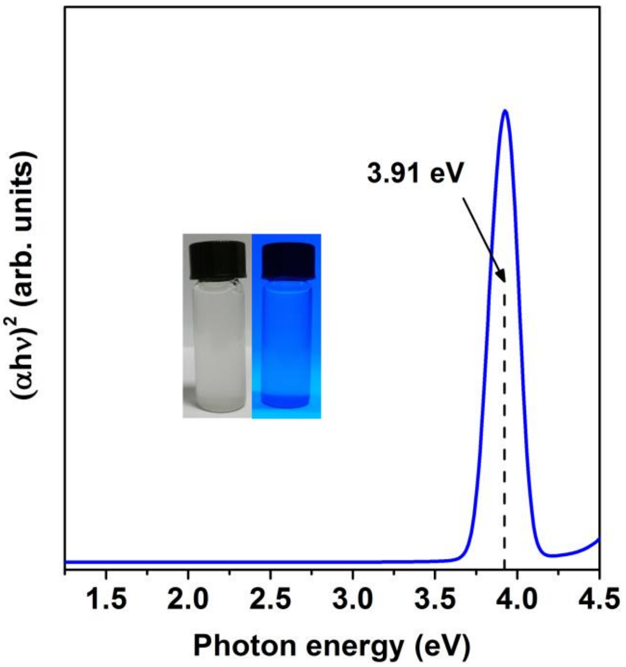

Supplementary Figure 1. The absorption band of Cs_4PbBr_6 NCs measured at ambient condition and the corresponding optical micrograph and PL photographs under UV irradiation.

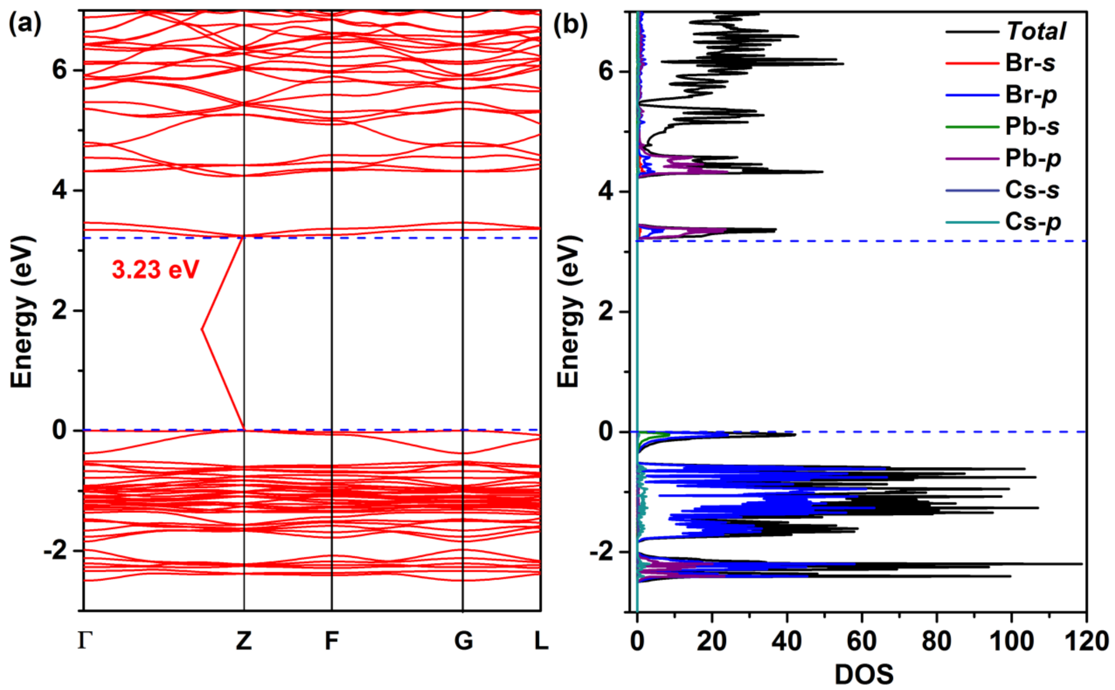

Supplementary Figure 2. (**a**) Calculated electronic band structure of Cs_4PbBr_6 perovskite at ambient condition. (**b**) Total and partial density of states projected onto the orbitals of Cs, Pb, and Br atoms. The spin-orbit coupling effect was included.

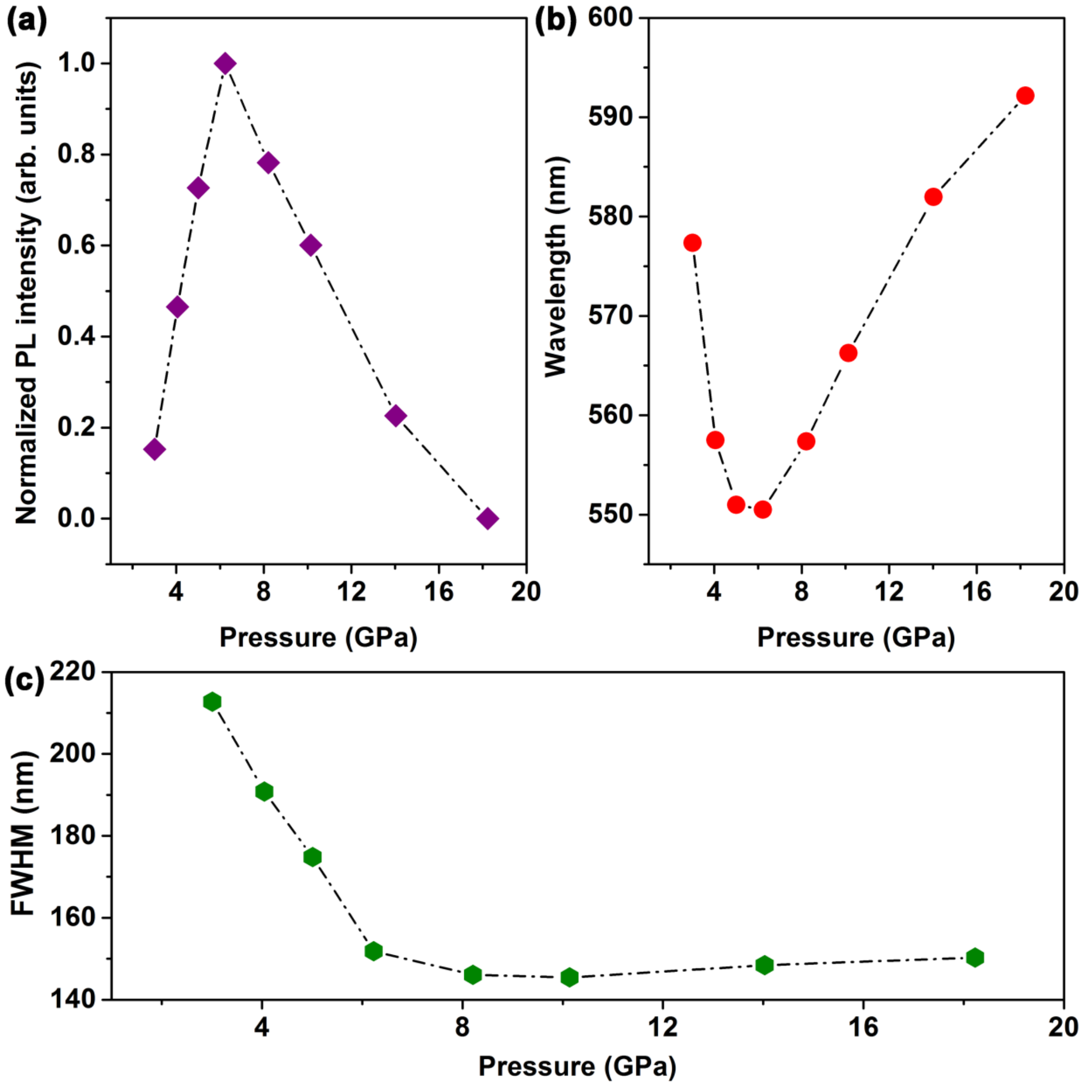

Supplementary Figure 3. (**a**) Normalized PL intensity as a function of pressure. (**b**) The PL location of Cs_4PbBr_6 NCs against pressures. (**c**) Full width at half-maximum (FWHM) vs pressure.

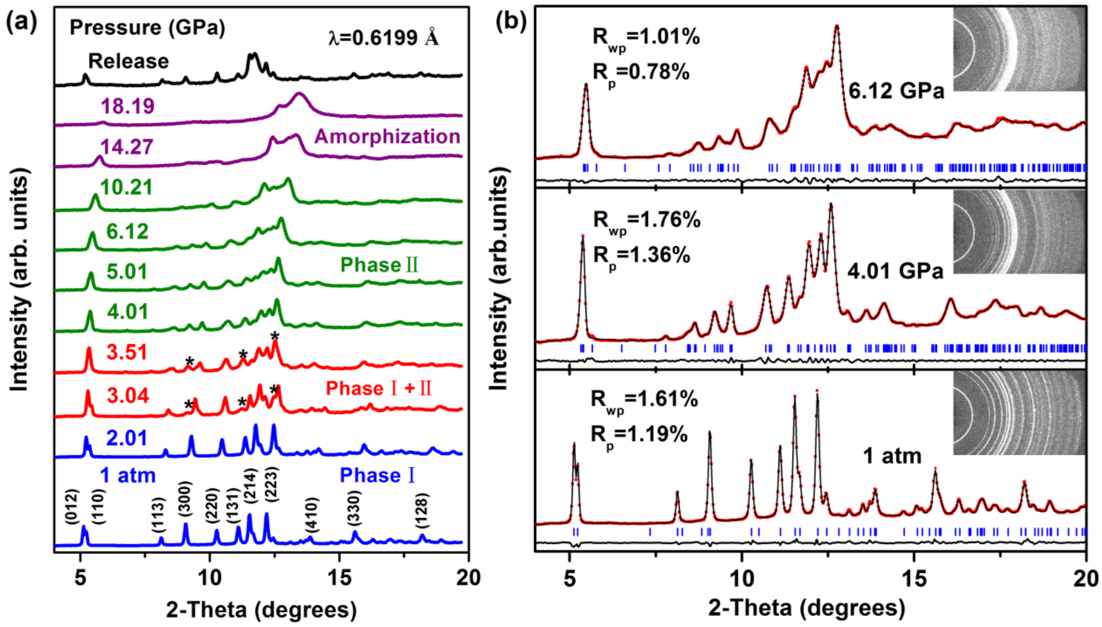

Supplementary Figure 4. (**a**) Representative in situ high-pressure XRD patterns of Cs_4PbBr_6 NCs with the presence of silicon oil as PTM during the high-pressure experiments. (**b**) Rietveld refinements of the experimental (red fork), simulated (black profile), and difference (black line) XRD patterns of phase I at ambient conditions and phase II at the pressure of 4.01 and 6.12 GPa. Therein, blue vertical markers indicate the corresponding Bragg reflections.

The in situ high pressure synchrotron ADXRD patterns of the Cs_4PbBr_6 NCs were collected with an incident monochromatic wavelength of 0.6199 Å (Supplementary Figure 4a). The ADXRD pattern at ambient condition confirmed that the initial structure showed good phase purity in the rhombohedral phase (denoted as Phase I). As the pressure increased, all the diffraction peaks of the Phase I shifted to higher angles due to a decrease in the volume of unit cell. With further compression, three distinct new peaks located in 9.15°, 11.21° and 12.46° appeared at 3.04 GPa, indicating the onset of a phase transition. At 4.01 GPa, the diffraction peaks assigned to the Phase I disappeared and the new peaks became dominant, suggesting that the phase transition was complete at that pressure point. At 6.12 GPa, the diffraction peaks underwent an apparent redistribution of intensities in 2θ range of 11° ~ 13°, which indicated that the transition involved substantial atomic rearrangements. Moreover, the broadening and the weakened intensity of diffraction peaks with pressure beyond 6.12 GPa manifested that the sample began to be disordered.[1] Almost all relative

strong diffraction peaks from the residual crystalline state disappeared upon compression to 18.23 GPa, corroborating the sluggish amorphous process of Cs_4PbBr_6 NCs under high pressure.[2] Supplementary Figure 4b shows the Rietveld refinement profiles of Cs_4PbBr_6 NCs at ambient pressure, 4.01 and 6.12 GPa. It was observed that the simulated XRD patterns by refinements agreed well with the experimental data. The refinement pattern of samples under ambient conditions confirmed that the initial structure is the rhombohedral phase with the space group of *R*-3*c*. The well-fitted XRD pattern at 4.01 GPa (R_P =1.36% and R_{WP} = 1.76%) can be readily indexed to the monoclinic structure in the space group of *B*2/*b* (Phase II). The refinement profiles at 6.12 GPa where the PL intensity reached the maximum, indicate that the structure remained the monoclinic lattice type with a lattice constant of a = 12.3832(4) Å, b = 9.4235(2) Å, c = 13.2333(3) Å, and β = 90.95(1) °.

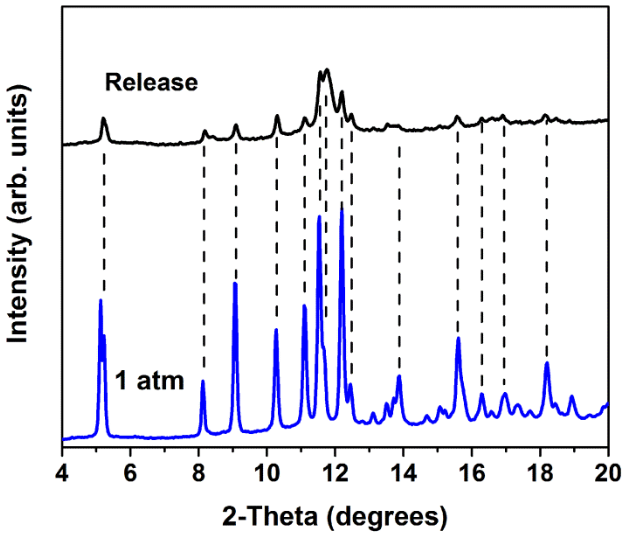

Supplementary Figure 5. Typical in situ ADXRD patterns of Cs_4PbBr_6 NCs at ambient condition (blue line) and quenched phase upon completely releasing the pressure to ambient conditions (black line). After decompression, the released ADXRD pattern retrieved its original rhombohedral structure again as depicted in the quenched profile.

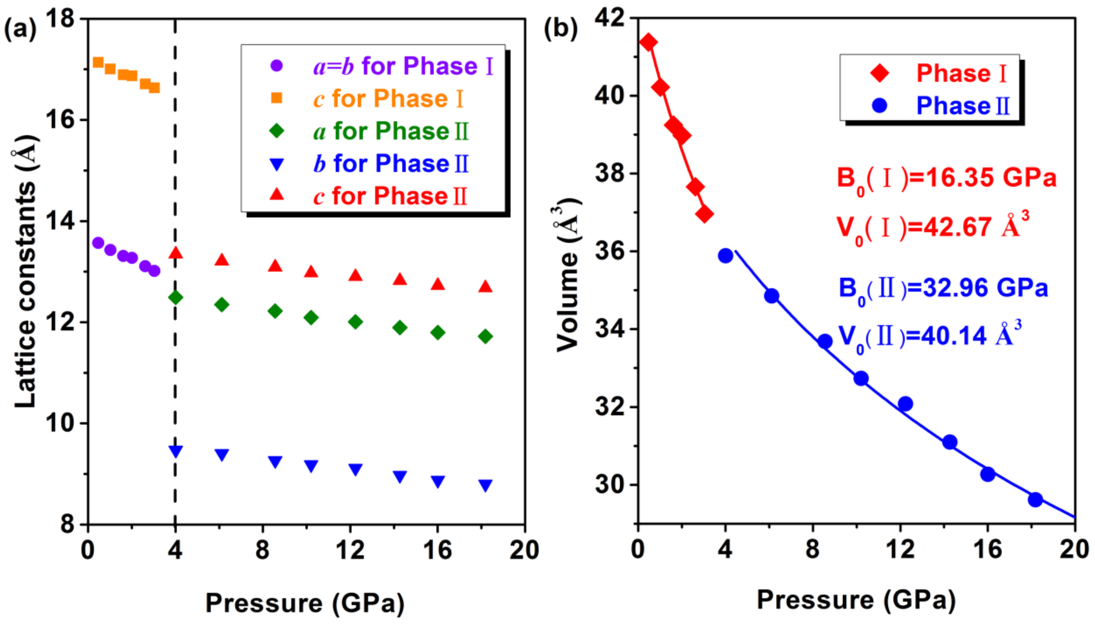

Supplementary Figure 6. (**a**) Lattice constants (*a*, *b*, and *c*) and (**b**) experimental volumes of Cs_4PbBr_6 NCs in Phase I and Phase II as a function of pressures up to 18.19 GPa. Detailed definitions of various symbols are interpreted in the figure. The solid lines represent the third-order fitted Birch-Murnaghan EOS functions to the measured P-V data.

We further conducted the pressure dependence of the lattice parameters and atomic volume change for phase I and phase II of Cs_4PbBr_6 NCs (Supplementary Figure 6). Apparently, the lattice parameters of phase II were less sensitive to external pressure, as compared with phase I. The experimental P-V data were fitted by the third-order Birch-Murnaghan equation:

$$\mathrm{P(V)} = \frac{3B_0}{2}\left[\left(\frac{V_0}{V}\right)^{\frac{7}{3}} - \left(\frac{V_0}{V}\right)^{\frac{5}{3}}\right]\left\{1 + \frac{3}{4}(B_0' - 4)\left[\left(\frac{V_0}{V}\right)^{\frac{2}{3}} - 1\right]\right\}$$

where V_0 is the zero-pressure volume, B_0 is the bulk modulus at ambient pressure, and B_0' is a parameter for pressure derivative. The isothermal bulk modulus B_0 for phase I and phase II were estimated to be about 16.35 GPa and 32.96 GPa, respectively. The much larger isothermal bulk modulus of phase II indicated highly difficult compressibility by contrast to phase I of Cs_4PbBr_6 NCs.

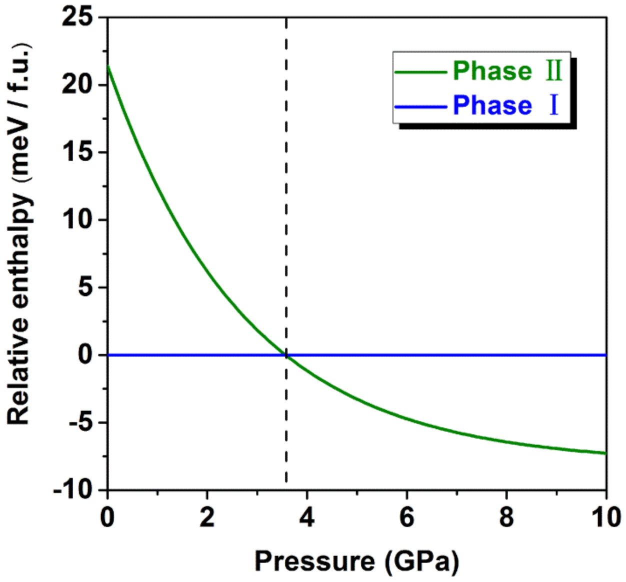

Supplementary Figure 7. Pressure dependence of calculated enthalpy per formula unit for the high-pressure monoclinic phase (Phase II) of Cs_4PbBr_6 relative to the ground-state rhombohedral structure (Phase I).

As depicted in Supplementary Figure 7, the first-principles energetic calculations of the high-pressure and ambient-condition phases of Cs_4PbBr_6 were performed based on the density functional theory (DFT). We note that in our calculations the high-pressure monoclinic phase (in the space group of *B*2/*b*, after structural optimization) consists of $[PbBr_6]^{4-}$ octahedra with two larger Pb-Br bonds and four smaller ones on the equatorial plane. The pressure dependence of the enthalpy difference between phase I and phase II indicated that the ambient-condition rhombohedral phase is indeed more stable under the lower pressures. Upon further compression, the high-pressure monoclinic phase was energetically more preferable beyond the pressure of ~3.6 GPa. This is in accordance with the experimental X-ray diffraction measurement.

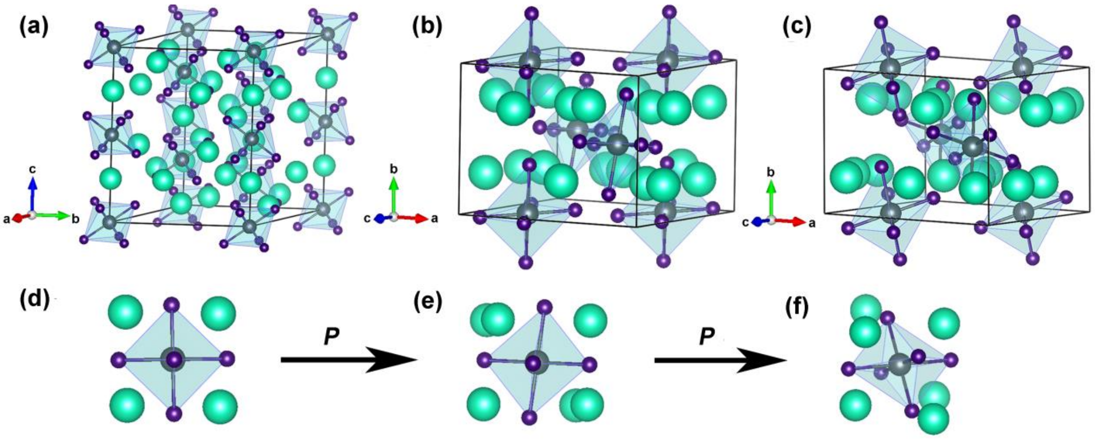

Supplementary Figure 8. Crystal structures and structure units of Cs_4PbBr_6 NCsat 1 atm (**a**, **d**), 4.01 GPa (**b**, **e**) and 6.12 GPa (**c**, **f**), respectively.

We further investigated the close correlation between the optical properties and the structural evolution of Cs_4PbBr_6 NCs upon compression. As the pressure increased in the phase I range, due to the decrease of the distance among the $[PbBr_6]^{4-}$ ions upon compression, the electrostatic interaction gradually gained its strength, which decreased the six bond lengths of Pb-Br within the regular $[PbBr_6]^{4-}$ octahedra (Supplementary Figures 8a, 8d). At around 3.04 GPa, the electrostatic interaction cannot compensate the increased free energy anymore. Therefore, the $[PbBr_6]^{4-}$ octahedra adopted another new configuration to reduce the free energy, leading to the occurrence of the phase transition in the range of 3.04 to 4.01 GPa. With further compression, the $[PbBr_6]^{4-}$ octahedral structure at Phase II began to undergo deformations gradually as shown in Supplementary Figures 8b, 8e. With further increasing the pressure, the electrostatic interaction is strengthened because the adjacent $[PbBr_6]^{4-}$ octahedra ions get closer, which induces the $[PbBr_6]^{4-}$ octahedra to be deformed persistently (Supplementary Figures 8c, 8f). However, when pressure exceeds 6.12 GPa, the distorted $[PbBr_6]^{4-}$ octahedra cannot afford the increased interaction. The $[PbBr_6]^{4-}$ octahedra will start to tilt and undergo a considerable rotation deviating from the original orientation in disordered way. As a consequence, the amorphous lattice appears.

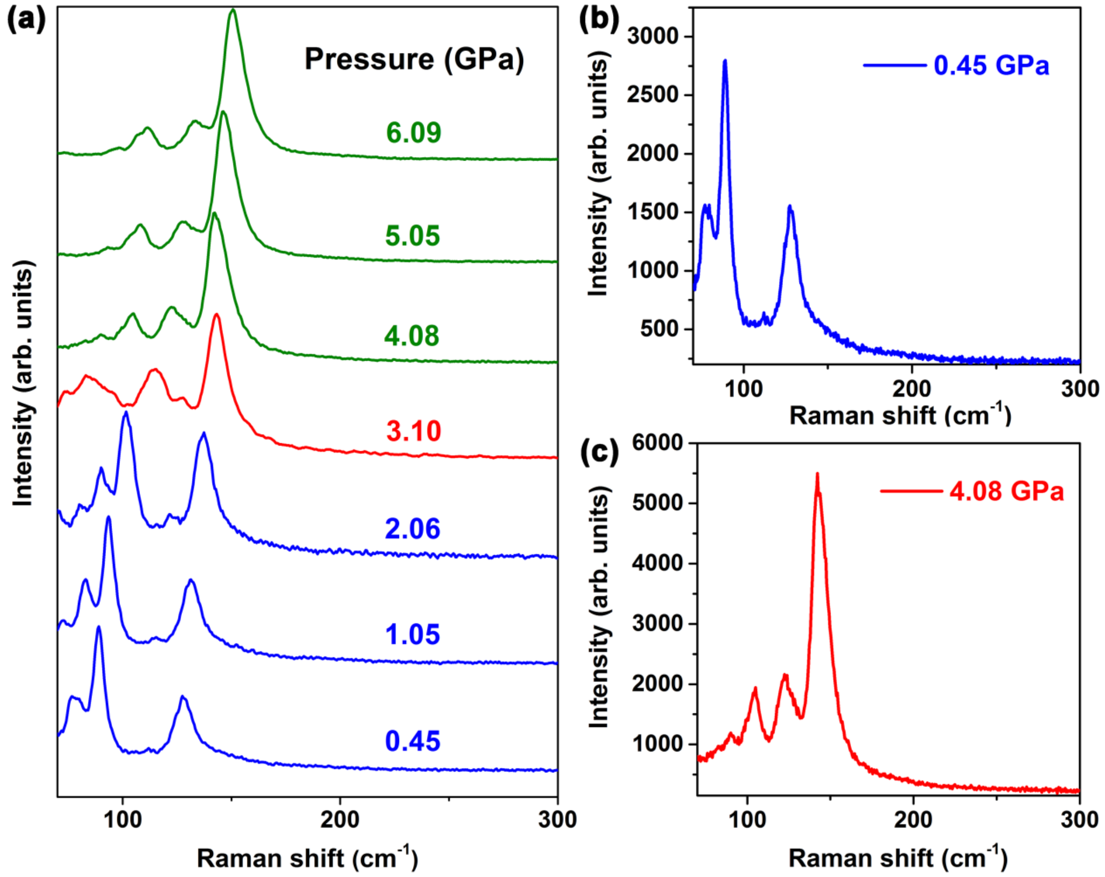

Supplementary Figure 9. (a) Selected Raman spectra of Cs_4PbBr_6 NCs at elevated pressure. (b) Raman spectra of Cs_4PbBr_6 NCs at 0.45 GPa. (c) Raman spectra of Cs_4PbBr_6 NCs at 4.08 GPa.

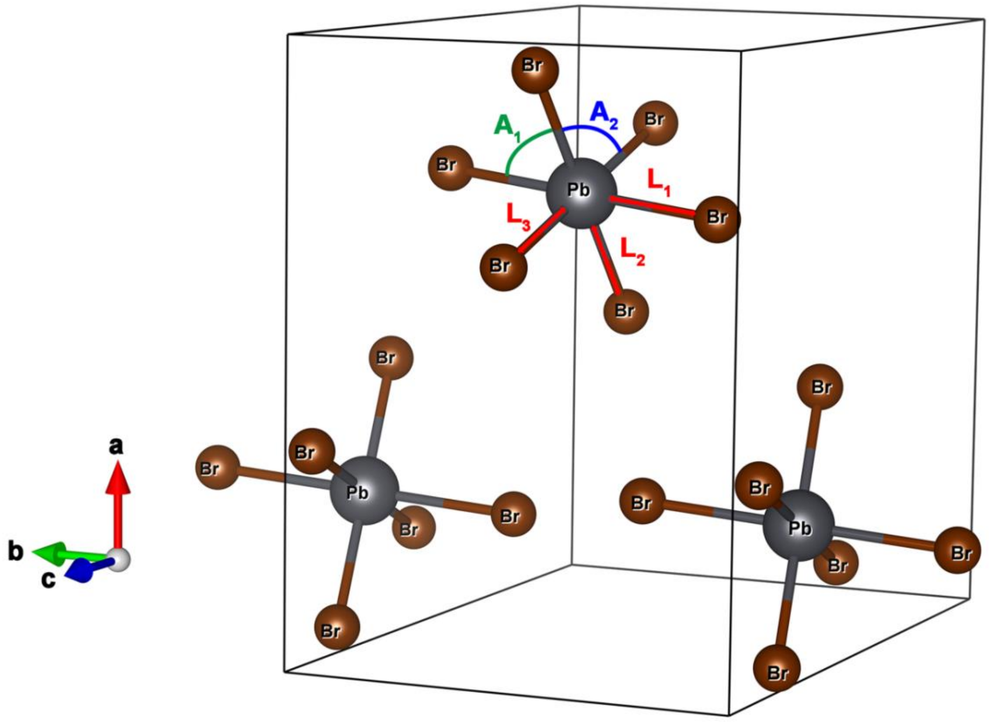

Supplementary Figure 10. Schematic diagram of the three non-equivalent Pb-Br bonds and two non-equivalent Br-Pb-Br angles within isolated $[PbBr_6]^{4-}$ octahedra for monoclinic Cs_4PbBr_6 crystals.

Supplementary Table 1. The three Pb-Br bonds and two Br-Pb-Br angles within isolated $[PbBr_6]^{4-}$ octahedra for Cs_4PbBr_6 crystal as function of pressure.

	**L_1 (Å)**	**L_2 (Å)**	**L_3 (Å)**	**A_1 (degrees)**	**A_2 (degrees)**
**1 atm**	2.989	2.989	2.989	90	90
**2.01 GPa**	2.925	2.925	2.925	90	90
**4.01 GPa**	2.891	2.767	2.845	86.043	90.578
**5.01 GPa**	2.89	2.766	2.852	85.824	90.292
**6.12 GPa**	2.881	2.761	2.856	85.506	90.142

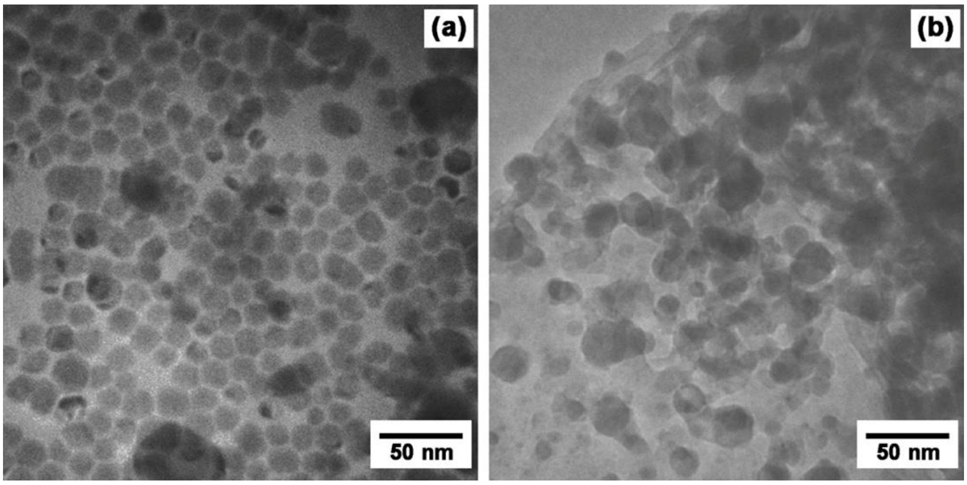

Supplementary Figure 11. TEM (a) images of the originally starting Cs_4PbBr_6 NCs. TEM (b) images of the decompressed Cs_4PbBr_6 NCs when the pressure was released to the ambient conditions.

The nonhydrostatic effect is an important factor for pressure-induced amorphization in perovskite materials.[3] As the loaded samples are NC aggregates, this possibly results in the generation of deviatoric stress across the Cs_4PbBr_6 NCs under higher pressures. Therefore, the nonhydrostatic effect cannot be excluded to interpret pressure-induced the weakening of PL. Above 6.8 GPa, all the diffraction peaks started to broaden and merge, likely due to the deviatoric stress induced amorphization. Additionally, the TEM image of the decompressed sample (Supplementary Figure 11) indicates a large aggregation and a high extent of deformation, which is a straightforward evidence for the above-claimed conclusion. Thereby, aggregation-resulted nonhydrostatic effect should be a crucial factor for the weakening of PL intensity.

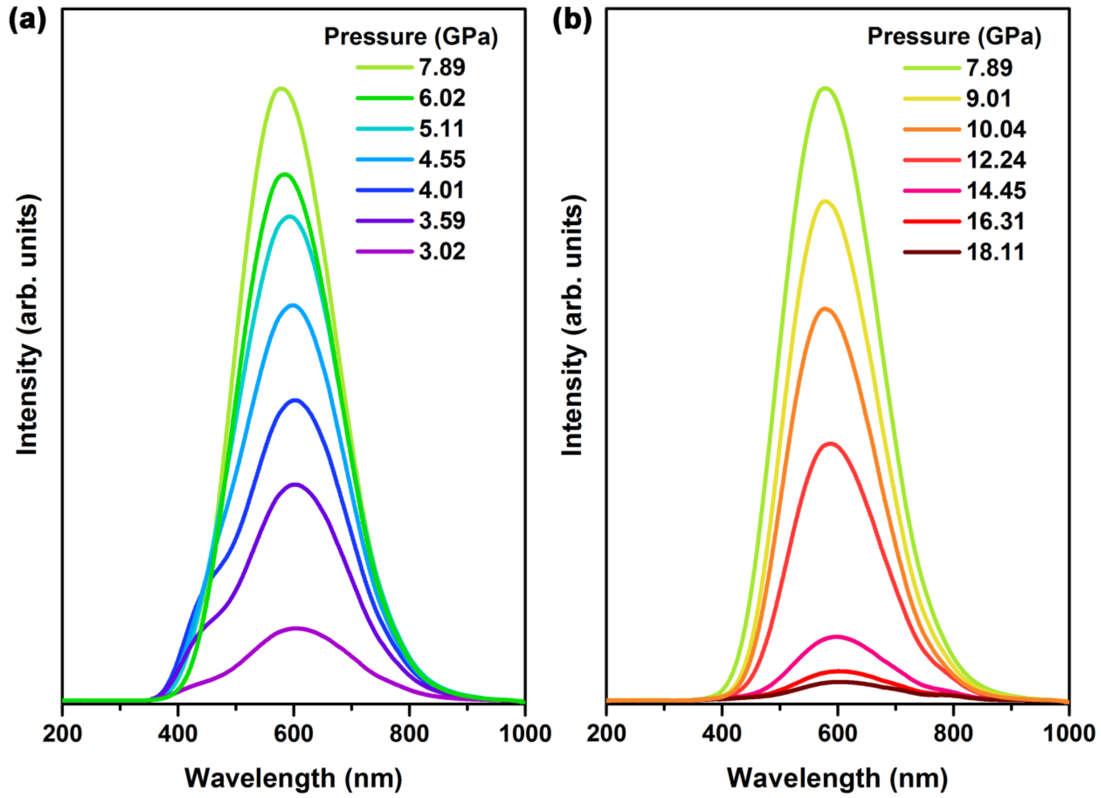

Supplementary Figure 12. Pressure-dependent PL spectra of Cs_4PbBr_6 NCs randomly dispersed in toluene.

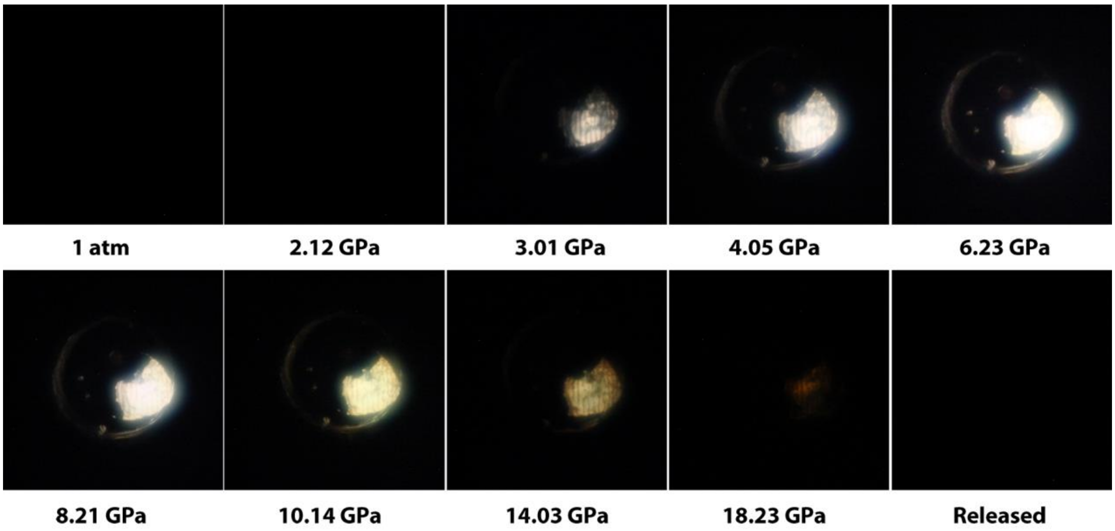

Supplementary Figure 13. In situ high-pressure PL photographs under UV irradiation (λ_{ex} = 355 nm) in a diamond anvil cell.

Supplementary Table 2. Chromaticity coordinates (CIE) and peak statistics at selected pressures for Cs_4PbBr_6 NCs.

Pressure (GPa)	CIE x	CIE y	Peak (nm)
3.01	0.3734	0.3852	576
4.05	0.3645	0.4010	559
5.01	0.3642	0.4157	549
6.23	0.3672	0.4321	549
8.21	0.3876	0.4483	558
10.14	0.4114	0.4574	566
14.03	0.4474	0.4595	581
18.23	0.4500	0.4397	592

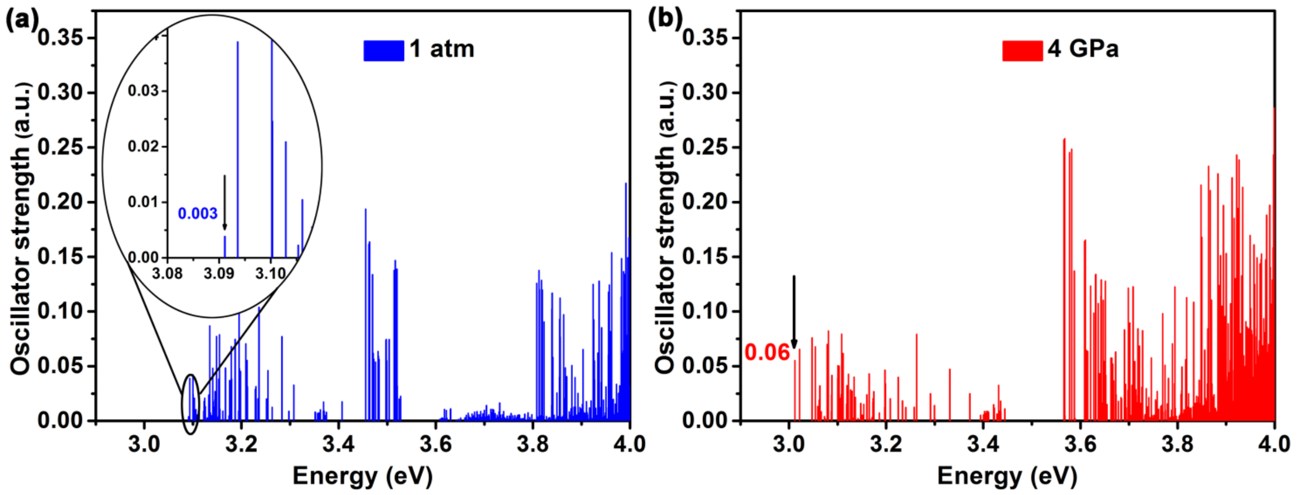

Supplementary Figure 14. Calculated absorption oscillator strengthes using the excited-state structure associated with self-trapped exciton at 1 atm (a) and 4 GPa (b), respectively.

The oscillator strengthes, *i.e.* transition dipole moments versus photon energy is shown in Supplementary Figure 14. We note that upon the appearance of the self-trapped exciton, the excited and ground states are many-body wavefunctions involving electron-hole pairs and lattice distortion (*i.e.*, phonon), therefore the strictly accurate calculation of transition dipole moments between them is complicated and challenging, which is beyond the scope of current study. For simplicity we took the excited-state structure associated with self-trapped exciton, *i.e.*, the lowest-energy spin-triplet state in company with lattice distortion, and calculate the transition dipole moments at the single-particle level. The calculated results, though approximated, are expected to capture the main feature of the realistic light emission process of self-trapped exciton. It should be also noted that the photon energies in the plots are not accurate in the single-particle approximation.

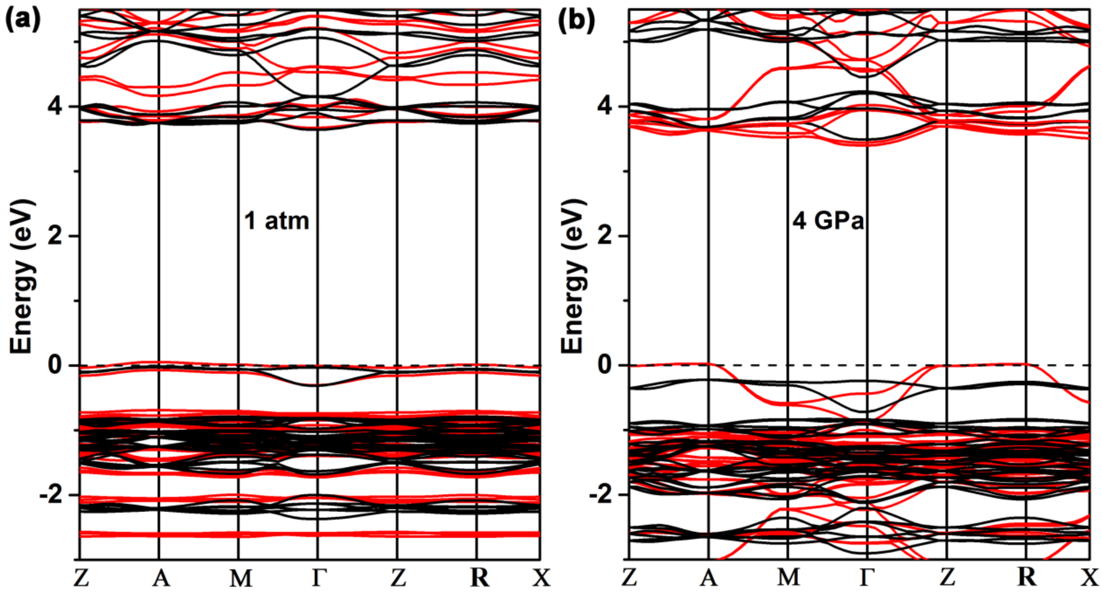

Supplementary Figure 15. Electronic band structure of Cs_4PbBr_6 with and without the lattice distortion mediating self-trapped exciton for the ambient-pressure (1 atm) and high-pressure (4 GPa) phases, respectively. The band structure of equilibrium structure (black lines) shifts upon the presence of lattice distortion (red lines). Note that the lattice distortion magnitude adopted in the calculations are the same for the ambient-pressure and high-pressure phases.

Supplementary References

1 Errandonea, D., Kumar, R. S., Manjón, F. J., Ursaki, V. V. & Tiginyanu, I. M. High-pressure x-ray diffraction study on the structure and phase transitions of the defect-stannite $ZnGa_2Se_4$ and defect-chalcopyrite $CdGa_2S_4$. *J. Appl. Phys.* **104**, 063524, (2008).

2 Wang, L., Wang, K. & Zou, B. Pressure-Induced Structural and Optical Properties of Organometal Halide Perovskite-Based Formamidinium Lead Bromide. *J. Phys. Chem. Lett.* **7**, 2556-2562, (2016).

3 Postorino, P. & Malavasi, L. Pressure-Induced Effects in Organic–Inorganic Hybrid Perovskites. *J. Phys. Chem. Lett.* **8**, 2613-2622, (2017).
